# The validity of synthetic clinical data: a validation study of a leading synthetic data generator (Synthea) using clinical quality measures

**DOI:** 10.1186/s12911-019-0793-0

**Published:** 2019-03-14

**Authors:** Junqiao Chen, David Chun, Milesh Patel, Epson Chiang, Jesse James

**Affiliations:** 1 Clinical Informatics, Evolent Health, Arlington, USA; 2 Health Administration and Policy, George Mason University, 4400 University Drive, Fairfax, Virginia 22030 USA

**Keywords:** Synthetic clinical data, Clinical quality measures, Model validation, Synthetic patient data

## Abstract

**Background:**

Clinical data synthesis aims at generating realistic data for healthcare research, system implementation and training. It protects patient confidentiality, deepens our understanding of the complexity in healthcare, and is a promising tool for situations where real world data is difficult to obtain or unnecessary. However, its validity has not been fully examined, and no previous study has validated it from the perspective of healthcare quality, a critical aspect of a healthcare system. This study fills this gap by calculating clinical quality measures using synthetic data.

**Methods:**

We examined an open-source well-documented synthetic data generator Synthea, which was composed of the key advancements in this emerging technique. We selected a representative 1.2-million Massachusetts patient cohort generated by Synthea. Four quality measures, Colorectal Cancer Screening, Chronic Obstructive Pulmonary Disease (COPD) 30-Day Mortality, Rate of Complications after Hip/Knee Replacement, and Controlling High Blood Pressure, were selected based on clinical significance. Calculated rates were then compared with publicly reported rates based on real-world data of Massachusetts and United States.

**Results:**

Of the total Synthea Massachusetts population (*n* = 1,193,439), 394,476 were eligible for the “colorectal cancer screening” quality measure, and 248,433 (63%) were considered compliant, compared to the publicly reported Massachusetts and national rates being 77.3 and 69.8%, respectively. Of the 409 eligible patients, 0.7% of died within 30 days after COPD exacerbation, versus 7% reported in Massachusetts and 8% nationally. Using an expanded logic, this rate increased to 5.7%. No Synthea residents had complications after Hip/Knee Replacement (Massachusetts: 2.9%, national: 2.8%) or had their blood pressure controlled after being diagnosed with hypertension (Massachusetts: 74.52%, national: 69.7%). Results show that Synthea is quite reliable in modeling demographics and probabilities of services being offered in an average healthcare setting. However, its capabilities to model heterogeneous health outcomes post services are limited.

**Conclusions:**

Synthea and other synthetic patient generators do not currently model for deviations in care and the potential outcomes that may result from care deviations. To output a more realistic data set, we propose that synthetic data generators should consider important quality measures in their logic and model when clinicians may deviate from standard practice.

## Background

Clinical data synthesis is an emerging technique that has the potential to boost clinical research, system implementation and training, while protecting patient privacy [1]. However, its validity has not been fully examined, which poses questions for its broader adoption.

Access to data is essential for research, implementation and training across disciplines. However, obtaining real-world data can be costly and often presents ethical challenges such as privacy concerns. This is particularly challenging in healthcare, where health records contain highly sensitive information and are strictly protected by laws and organizational policies [1].

To circumvent these challenges, some organizations and individuals have developed different approaches to synthesize clinical data. These approaches are usually based on some probability-based logic and completely bypass the use of real patient-level data. By doing so, it imposes no risk for revealing personally identifiable information. Example products include Patient Generator [2], EMRbots [3], and Synthea [1]. Synthea, for example, emphasizes the use of publicly available health statistics (e.g., census) and clinical guidelines, and attempts to make the synthetic data sufficiently realistic but not real [1].

Before wider use of synthetic data, its validity needs to be tested, e.g. how closely synthetic data is equivalent to real data [4]. Researchers in this field usually referred to this property as the “realism” of synthetic data [5], and researchers in the broader simulation community called it as operational validity and the process of assuring this property as operational validation [6], which consists of a variety of methods. A recent article [5] found that there was no consensus on the methods most appropriate for operational validation of synthetic clinical data, and only few studies have actually done it. This is not a unique shortcoming of synthetic clinical data. Earlier review found that validation of healthcare simulation in general was lacking [6, 7], which consists of a variety of methods.

Because quality of care is one of the primary goals and characteristics of a healthcare system [8], we consider it critically important to have synthetic data presenting the same level of care quality as real data. Therefore, we will use clinical quality measures to validate the synthetic clinical data. Quality measures are evidence-based metrics to quantify the processes and outcomes of healthcare. They are widely used to indicate the level of effectiveness, safety and timeliness of the services that a healthcare provider or organization offers [9].

After reviewing the three synthetic data products, we decided to focus on Synthea because it is open-source, well-documented in peer-reviewed journal articles and online documentation. Patient Generator is a commercial product that builds its core modules based upon Synthea, so our understanding of Synthea would largely apply to Patient Generator. We determined EMRBots as ineligible for our study because, as described later, most of the quality measures chosen focus on health outcomes, which is not an aspect that EMRBots considers in its design. According to the creator of EMRBots, this is because it doesn’t model time-dependent interactions between patient factors and clinical outcomes [3]. Synthea models care processes after clinical guidelines and models care outcomes after literature and clinical expertise. Synthea currently models 38 clinical conditions and their progressions; simulating patient-provider encounters, lab data, medication prescription and more [1]. In this aspect, we find Synthea to be the most comprehensive, open-source synthetic patient generator that is freely available for our validation study. Quality measures might be effective to uncover some unrealistic aspects of Synthea because Synthea models mainly after clinical guidelines, which describe what ideally should happen, while quality measures are “the other side of the coin” to spotlight suboptimal care. So far, although it has been suggested [10], quality measures have never been used in the existing few validation studies [5]. We present the first study using this method.

By doing this study, we hope to contribute to the healthcare community from three perspectives. From the perspective of synthetic data developers, we hope to provide an external validation on a representative product, shedding lights on potential areas of improvement. From the perspective of synthetic data users (researchers, system implementers, teachers, health policy developers), we hope to provide some insights on for what use cases that synthetic data would be a reliable replacement of real data and for what use cases it is not. Lastly, from the perspective of the broader healthcare community, we argue that improving a general-purpose synthetic data generator such as Synthea would essentially improve our understanding of how the healthcare system works. As mentioned above, healthcare quality is an important pillar of a healthcare system. To explain any difference between the quality scores derived from synthetic data and the ones from real-world data could help us better understand the contributing factors to real-world healthcare quality.

## Methods

### The SyntheticMass dataset

The dataset we used is called SyntheticMass, which contains more than one million “synthetic residents” of Massachusetts pre-generated using Synthea and ready for free download [11]. The goal of this synthetic population was to statistically mirror the Massachusetts population regarding demographics, disease burdens, vaccinations, medical visits and social determinants [11]. To achieve this goal, the Synthea model was initiated by real demographics data of Massachusetts residents on the census track, town and county levels. Demographic variables included population, percentage of difference races, median age, median household income, and percentage of college graduates. After the synthetic patients was created, they went through their clinical journeys per disease modules. Explained in detail below, a disease module essential simulates patients’ through a series of clinical processes per recommendations from clinical guidelines and projects their care outcomes per findings from literature or input from clinical experts. If a disease module is set up correctly, it should imitate real-world health care phenomena, including the quality of care.

We found this population to be the most appropriate for our study because of two reasons. Firstly, it attempts to mimic the characteristics of the entire population of Massachusetts, which would make our quality measure results comparable to those that are publicly reported. Secondly, because Synthea adopts Monte Carlo simulation technique, it generates a slightly different population every time the software is run [10]. Using a large, representative, pre-generated population on the other hand, would facilitate other researchers to replicate our work.

SyntheticMass dataset contains a series of tables to mimic typical information from an electronic health record system. Within these tables, we mainly focused on the “encounter”, “condition” and “patient” tables. The encounter table entails patients’ encounters to health facilities, such as the service date, encounter type and principal diagnosis. The condition table provides information on onset and end dates for clinical conditions (signs, symptoms and diagnoses). The condition table accumulates all identifiable conditions that a patient has, even a condition is not the main reason why a patient seeks care in a particular encounter. The patient table provides demographic information such as identifiers, address, birth date, death date (if applicable), and gender. Modeled after electronic health records, diagnoses and procedures in Synthea are coded using Systematized Nomenclature of Medicine -- Clinical Terms (SNOMED-CT).

### Disease modules and quality measures

We started with selecting quality measures relevant to the clinical modules available in Synthea. Then we obtained the publicly reported rates of the selected measures for Massachusetts and United States as our real-world reference. We then obtained the specifications of those measures and calculated rates using SyntheticMass datasets, so that we could compare the results from real data to those from synthetic data.

**Fig. 1:**
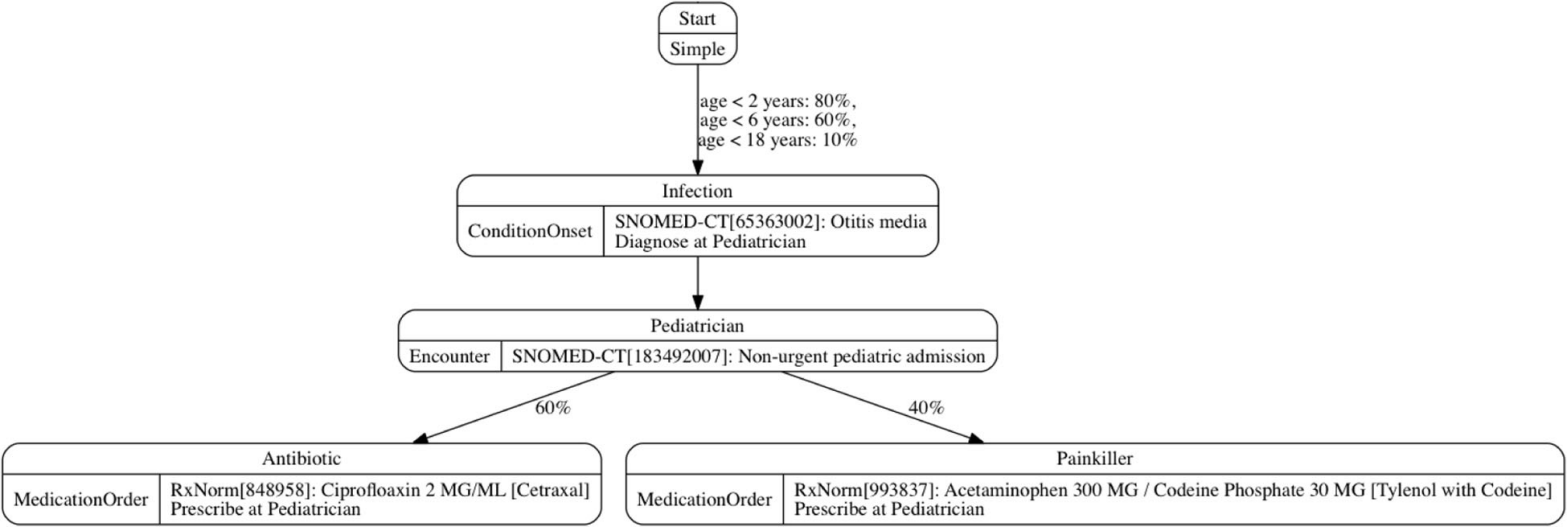
An example disease module reproduced from [26] with permission to use from [1]

A clinical module is the basic unit in Synthea to model “clinical” and “control” events (or “states” in technical terms) in a clinical domain. “Clinical states” effect disease progression and care, while “control states” effect flow control. Figure 1 is a simplified example of children ear infection provided by Synthea. Children have varying likelihoods of developing ear infection based on their age, which then triggers a non-urgent pediatric admission. During the admission a patient has certain chance of taking either an anti-biotic or painkiller. The example stops here but for other modules, there is usually a process to model outcomes after the treatment (e.g., certain chance of recovery). However, as discussed later, the modeling of outcomes might be indeed a shortcoming in Synthea. Currently there are 38 modules in Synthea, ranging from allergies, chronic diseases (e.g. Asthma), to social circumstances (e.g. homelessness).

**Table 1:** Information on Selected Measures

Measure Name	Eligibility Criteria	Type of Measure	Reporting Organizations	Related Synthea Modules
Colorectal Cancer Screening	Patients between 50 and 75 years old who have had a colorectal cancer screening in a specified time frame (time frame dependent on last test taken).	Process measure	Centers for Medicare and Medicaid Services	Colorectal Cancer
COPD 30-day mortality	Patients who have had an admission with principal diagnosis of COPD exacerbation and died within 30 days of index admission.	Outcome measure	Centers for Medicare and Medicaid Services	COPD
Complications after Hip/Knee Replacement	Patients who have had either Total Knee Arthroplasty or Total Hip Arthroplasty and have had a complication within a specified time frame (time frame dependent on complication).	Outcome Measure	Centers for Medicare and Medicaid Services	Total Joint Replacement
Controlling High Blood Pressure	Patients diagnosed with hypertension that have had their blood pressure at or below the target blood pressure in subsequent blood pressure readings.	Outcome Measure	National Quality Assurance Committee	Metabolic Syndrome Disease

Our selection criteria was that a quality measure needed to correspond to a clinical module available in Synthea, and was also publicly reported in quality reporting programs. After reviewing measures in the Healthcare Effectiveness Data and Information Set (HEDIS), Hospital Compare and Star Ratings, we found four measures eligible with Synthea’s modules: Colorectal Cancer Screening, Chronic Obstructive Pulmonary Disease (COPD) 30-Day Mortality, Complications after Hip/Knee Replacement, and Controlling High Blood Pressure. Table 1 provides information on each of the selected measures. HEDIS is operated by the U.S. National Quality Assurance Committee (NCQA) and is collection of performance measures geared towards health insurance plans [12]. Hospital Compare [13] is a website operated by the U.S. federal agency Centers for Medicare and Medicaid Services (CMS) that provides performance data from participating hospitals. Star Ratings is also operated by CMS and is a rating tool that uses mostly health insurance claims data to grade Medicare Advantage plans based on quality measures and other metrics [14]. They are both public programs operated by the U.S. federal agency Centers for Medicare and Medicaid Services designed for patients to be able to compare hospitals and health plans in their area with others. Although Hospital Compare and Star Ratings use a variety of data sources, the measures that we selected for this study all use administrative claims data [15]. These measures correspond to the Colorectal Cancer, COPD, Total Joint Replacement, and Metabolic Syndrome Disease Modules in Synthea [16]. The clinical significance of each measure will be elaborated below. As a set, they cover both preventive service and chronic disease management, both ambulatory care and hospital care, and both medical service and surgical service. The first quality measure we calculate using synthetic data is Colorectal Cancer Screening, which requires patients aged 50 to 75 to have appropriate screening for colorectal cancer. This measure is important because treatment for colorectal cancer in its earliest stage can lead to high survival rate, colorectal cancer screening for adults in the 50–75 age group can help detect potentially cancerous polyps or colorectal cancer early [17]. We included patients who were alive in 2015 and 2016. Five tests were considered appropriate in this measure: Colonoscopy (recommended every 10 years), Flexible Sigmoidoscopy (every 5 years), Computed Tomography Colonography (every 5 years), Fecal Occult Blood Test (every year), and Stool DNA Test (every 3 years).

### COPD 30-day mortality

The COPD 30-day mortality measure is defined as patients who died during or within 30 days of index admission with a principal diagnosis of COPD exacerbation. This measure is important because patients hospitalized after COPD exacerbations have had their mortality rate significantly affected by the quality of care given. Mortality is used because it is an indicator of the overall efficacy of more difficult to measure individual processes [17].

As shown later in the result section, the denominator identified by strictly following the measure specification is small. For sensitivity analysis, we expanded the definition and examined how that might influence the result. The strictly-defined calculation only used encounters with a principal diagnosis of COPD as part of the denominator/numerator criteria. The expanded calculation included other encounters where the COPD condition was active at the time of the encounter date.

### Complication rate for hip/knee replacement

This measure looks for the occurrence of 8 complications within specific time periods after the hip or knee replacement surgery. With an aging population with high rates of osteoarthritis, Total Hip Replacement and Total Knee Replacement complications have been identified as a priority area for outcome measure development [18]. Heart attack, pneumonia, sepsis, septicemia or shock would be counted in numerator if it happens within 7 days of the admission; surgical site bleeding, pulmonary embolism, or death within 30 days; or mechanical complications, periprosthetic joint infection or wound infection within 90 days of admission. The index admission date is defined as the encounter date with one of two SNOMED-CT codes: Total Knee Replacement (609588000) and Total Hip Replacement (52734007).

### Controlling high blood pressure

The measure for controlling high blood pressure looks for patients with a diagnosis of hypertension who have had their subsequent blood pressure measurements below 140/90 mmHg (for patients aged 18–59 or 60–85 with diagnosis of diabetes) or 150/90 mmHg (for patients aged 60–85 with no diagnosis of diabetes). This measure is important for population health because hypertension increases a patient’s risk for heart disease and stroke, both of which are leading causes of death in the United States [19]. In this calculation, we found patients in the synthetic population with an active condition of hypertension (SNOMED-CT: 38341003), and have had their blood pressure adequately controlled to below the defined limits after the condition onset date.

### Calculated rate using Synthea

A quality measure consists of value sets and clinical logic. A value set is a list of medical codes used to determine numerator and denominator eligibility for the selected measures used value sets, a set of medical codes used in administrative claims and electronic medical records to define diagnoses and procedures, to identify numerator and denominator eligibility for each clinical quality measure. For example, a value set for influenza may contain ICD-10 codes that are related to an influenza diagnosis. The value sets for each quality measure are defined in the technical specifications of each measure, and were obtained from the Value Set Authority Center (VSAC). Mapping between coding systems or terminologies is necessary because these measures were mostly developed for administrative data, thus International Classification of Diseases Version 10 (ICD-10) and Current Procedure Terminology (CPT) was used to codify diseases and procedures, respectively. The ICD-10 and CPT codes were mapped to SNOMED-CT in accordance to documentation obtained from VSAC. When needed, we mapped ICD-10 or CPT codes to SNOMED-CT, which, as mentioned above, is the terminology used in Synthea.

After obtaining quality measure compliance rates for the SyntheticMass population, we carried out statistical bootstrapping in SPSS (IBM Armonk, New York) with 1000 resamples to obtain a 95% confidence interval for each measure.

### Publicly reported rate

For the COPD 30-Day Mortality measure and Complications after Hip/Knee Replacement measure, we used publicly reported rates found on Hospital Compare as the reference population for comparison. For the Colorectal Cancer Screening and Controlling High Blood Pressure measure, the real-world rates of Massachusetts were aggregated from publicly reported Star Rating data of nine health insurance companies based in Massachusetts. National measure data was obtained by using rates from the 2017 Healthcare Effectiveness Data and Information Set report [20, 21].

## Results

As shown in Table 2, SyntheticMass has a population size of around one sixth of the real Massachusetts population. Apart from average weight and BMI, the synthetic population has comparable demographic characteristics as the Massachusetts population. The likeness of demographic data between the synthetically generated population and Massachusetts population might be attributed to the use of the real-world population as a reference for calibration when developing the Synthea’s data generation process. However, we observed that the SyntheticMass population is much more obese (BMI) on average compared its real-world counterpart.

**Table 2:** Comparison of Demographics between SyntheticMass and Real World Populations [27–29]

	SyntheticMass	Reference Population Estimates
Total Population	1,193,439	6,547,629
Age		
< 18	19.2%	21.7%
18–64	64.5%	64.5%
≥ 65	16.3%	13.8%
Average	40	39.1
Female	51%	51.6%
BMI (Adult)		
< 18.5	2%	N/A^b^
18.5–24.99	15.4%	N/A^b^
25–29.99	20%	35.6%
≥ 30	62.6%	23.3%
Male Average	31	28.7
Female Average	32	29.2
Average Height (cm)		
Male	176.77	175.77^a^
Female	163.33	161.80^a^
Average Weight (kg)		
Male	97.98	88.77^a^
Female	86.04	76.43^a^
Adults ≥20 years old with High Blood Pressure	30.92%	33.4%

^a^Nationally Reported Rate, all other rates shown reflect Massachusetts population

^b^No publically reported data found for reference

Our calculation for Colorectal Cancer Screening identified 314,355 eligible members (denominator), with 215,919 of them numerator compliant (68.7%). This is below the average for Massachusetts population (77.3%) but closer to the nationally reported rate (69.8%). An interesting observation is that Synthea only modules two out of the five eligible tests in their modules: Colonoscopy and Fecal Occult Blood Test.

For the COPD Mortality 30-day measure, our calculation under strictly-defined specification returned a total of 409 encounters with a principal diagnosis of COPD during the measurement year of 2016. Of the 409 eligible admissions, three had associated patient deaths within 30 days of the index admission date (0.7%). With the concern that this logic might be too stringent, we expanded the inclusion criteria to include all admissions from patients with COPD related conditions. Using the expanded specification, the number of encounters in the denominator increased significantly to 181,458, of which 10,373 deaths occurred within 30 days. The expanded rate for this measure is 5.7%, which is lower than the national rate of 8% and state average for Massachusetts hospitals, 7% (1233/17636, range 5.2–9.3%). Another interesting observation is that only two SNOMED-CT codes that are related COPD were used in Synthea: 185086009 (Chronic Obstructive Bronchitis) and 84,733,001 (Pulmonary Emphysema), compared to the 20 ICD-10 diagnosis codes included in the Hospital Compare value sets, Synthea might not be sufficient to describe all the different types and nuances of COPD.

Our Hip/Knee complication measure identified zero patients who met the numerator criteria. Within the entire SyntheticMass population, only 207 synthetic patients had a hip/knee replacement during the measurement period of 2016 (denominator). None of them had an admission with heart attack or pneumonia 7 days after the procedure; or died within 30 days after the procedure. Even after expanding parameters to include any patients who had a condition onset date 7 or 30 days after procedure, our calculation yielded no patient for numerator. The Massachusetts average rate calculated from real data is 2.92% (700/23949, range 1.9–4.4%). Although 0 and 2.92% is a small difference arithmetically, it triggers serious concern over Synthea’s capability to model postoperative complications. A potential explanation is that of the 8 complications, there was only one code (Pneumonia, SNOMED-CT 233604007) directly being used in Synthea. We tried every effort to identify these complications using different codes, and did try to use Myocardial Infarction (SNOMED-CT 22298006) to replace Acute Myocardial Infarction in the original specification, we still could not identify even one numerator case.

As shown in Table 3, the Colorectal Cancer Screening rate of SyntheticMass is very close to the national rate. This is expected since this measure is a procedure-based measure, and Synthea’s strength is exactly that it could model the probability of certain services offered in different phases of care. However, it is much lower than the Massachusetts rate. This infers that Synthea could not model regional variation in quality. The other two outcome-based measures, on the other hand, are much more complex to model as there are many factors involved in the services that may impact the outcomes. Even with expanded logic, the rates of SyntheticMass are still much lower than Massachusetts or national rates.

**Table 3:** Quality Measure Rates of SyntheticMass versus State/National Rates

Measure	SyntheticMass Rate	Massachusetts Rate	National Rate
Colorectal Cancer Screening	68.7% (68.5, 68.9%) (215,919/314,355)	77.3%	69.8%^a^
COPD 30-Day Mortality: Strict	0.7% (0, 1.7%) (3/409)	7.0% (1233/17636)	8.0%
COPD 30-Day Mortality: Expanded	4.7% (4.6, 4.8%) (8612/181,458)	7.0% (1233/17636)	8.0%
Complications of Hip/Knee Replacement	0% (0/207)	2.9% (700/23949)	2.8%
Controlling High Blood Pressure	0% (0/241,311)	74.52%	69.7^a^%

Numbers in parentheses represent lower and upper limits for 95% confidence interval, respectively

^a^Medicare PPO Reported Rate: Measure reported rates not available from HospitalCompare, rate acquired from HEDIS Medicare PPO rate. HEDIS reports rates for Medicare PPO and HMO, Medicaid PPO, Commercial PPO and HMO. Reported compliance rates for Colorectal Cancer Screening range from 58.3% to 69.8 and for Controlling High Blood Pressure range from 54.5 to 69.7%

Our measure for Controlling High Blood Pressure resulted in zero patients meeting numerator criteria. Of the total SyntheticMass population, 241,311 synthetic adult patients had hypertension (29.91%). We hypothesized that a certain percentage of hypertensive could have their blood pressure controlled post treatment. After analyzing Synthea’s data, this doesn’t seem to be the case. This may indicate that although Synthea is modeled to simulate a realistic percent of population with hypertension, it does not realistically model the outcomes that may occur post-diagnosis of hypertension as a result of intervention.

## Discussion

In this paper, we attempt to validate the realism of Synthea, a synthetic clinical data generator, by calculating clinical quality measures and comparing the results with real-world rates.

Our analysis shows that, apart from average weight and BMI, the synthetic population has comparable demographic characteristics as the Massachusetts population. We speculate two reasons behind this. Firstly, similar to the issue of hypertension (once a patient becomes hypertensive, the condition controlled), once a patient becomes obese, he or she might not lose weight, which inflates the overall obesity rate in the entire population. Secondly, it could also be due to the lack of reference data to accurately calibrate the model, or a difficulty in simulating height/weight interactions simultaneously to create an accurate BMI distribution.

Results of validation using quality measures indicate Synthea has both strengths and weaknesses in its approach. On one hand, as evident in the Colorectal Cancer Screening result, Synthea presents a high level of reliability in modeling the probabilities of certain services being offered in an average healthcare setting. On the other hand, as evident in other outcomes measures and a variance analysis between hospitals, its capabilities to model heterogeneous post-intervention health outcomes limited. We are inconclusive on Synthea’s possible limitations in modeling for regional variances in quality, due to the little variance between the national and state reported rates for all measures but Colorectal Cancer Screening. This is indeed a difficult task, testified by creators of another synthetic data generator EMRBots [3].

This is the first study that uses quality measures to validate synthetic data. Results highlight the importance of incorporating quality measures in the synthesis logic, which, as far as we know, has not been considered in any existing products. The assumption that all care processes will follow clinical guidelines is not realistic. In real-world clinical practices, noncompliance with clinical guidelines is very common and diverse variations in healthcare utilization have been observed for decades [22]. In the long term, an ideal synthesis logic should account for all the factors that influence compliance with clinical guidelines, including the guideline’s quality itself, clinicians’ attitude to behavioral changes, an organization’s resources and many more [23]. Quality measures could serve as one way to explore and verify our understanding of these factors. Such a thinking process could also apply to other aspects of realism in synthetic data besides “quality”, such as “cost” or “access”.

This leads to an important viewpoint on the contribution of synthetic data in general. It is not merely creating another source of data we could safely play with. By researching all the underlying mechanisms that could increase its realism, we could gradually parameterize the factors and interactions that make our health system the way it is now. Quoting Epstein’s famous article *Why Model?* (Epstein, 2008, [24]), the development and calibration of a simulation model could offer explicit explanation of real-world phenomena, guide data collection, illuminate core dynamics, raise new questions and more. All of these are critical to enhance our understanding of the complexity in health care [25].

Our study has a few limitations. Firstly, we could only identify publicly reported quality measures for four clinical modules in Synthea. This might undervalue Synthea, which might present higher realism in other, less complicated, modules. Secondly, for the selected clinical modules, we only had one quality measure each for validation, which is not optimal since “quality” is a multi-faceted concept. However, the quality measures examined in this paper have been widely adopted in national quality programs and represent fundamental facets of quality in those clinical domains. We believe they are the basic ones that Synthea needs to model after to improve its realism. Thirdly, the original specification of these quality measures are designed mostly for administrative data, which is different from the clinical data (electronic health records) that Synthea generates. Although this may have an impact on the calculated rates, the differences between the synthetic rates and real-world rates are so big for three measures that we believe it could not be solely attributed to the features of different data sources.

## Conclusion

In order to spread the use of synthetic clinical data, its realism needs to be tested. Clinical quality measures could serve as an effective validation tool because it is critical that synthetic data presents the same level of care quality as real data. After applying quality measures in Synthea, its strength and weakness have been uncovered, especially its limited capability to model heterogeneous health outcomes after major interventions. To improve its realism, Synthea and other synthetic data generators needs to model factors that make clinical practice deviate from standard guidelines and introduce variations in healthcare quality. Doing so could contribute to our overall understanding of the complexity in healthcare. Future validation studies should continue to identify eligible quality measures to validate new modules available in Synthea, and identify publicly reported rates based on electronic medical records. If Synthea and other synthetic data generators could be continuously improved, expanded and rigorously validated with variations in health care quality in mind, we are optimistic about the future of synthetic clinical data.

## Abbreviations

CMS: Centers for Medicare and Medicaid Services
COPD: Chronic Obstructive Pulmonary Disease
CPT: Current Procedural Terminology
HEDIS: Healthcare Effectiveness Data and Information Set
ICD-10: International Classification of Diseases and Related Health Problems, revision 10
SNOMED-CT: Systematized Nomenclature of Medicine -- Clinical Terms
VSAC: Value Set Authority Center
